# The Effect of *Cantharellus Cibarius* Addition on Quality Characteristics of Frankfurter during Refrigerated Storage

**DOI:** 10.3390/foods8120635

**Published:** 2019-12-03

**Authors:** Sasa Novakovic, Ilija Djekic, Anita Klaus, Jovana Vunduk, Vesna Djordjevic, Vladimir Tomović, Branislav Šojić, Sunčica Kocić-Tanackov, Jose M. Lorenzo, Francisco J. Barba, Igor Tomasevic

**Affiliations:** 1 Faculty of Agriculture, University of Belgrade, 11080 Belgrade, Serbia; idjekic@agrif.bg.ac.rs (I.D.); aklaus@agrif.bg.ac.rs (A.K.); vampum00@yahoo.com (J.V.); 2 Institute of Meat Hygiene and Technology, Kaćanskog 13, 11080 Belgrade, Serbia; 3 Faculty of Technology Novi Sad, University of Novi Sad, 21000 Novi Sad, Serbia; tomovic@uns.ac.rs (V.T.); bsojic@gmail.com (B.Š.); suncicat@uns.ac.rs (S.K.-T.); 4 Centro Tecnológico de la Carne de Galicia, Parque Tecnológico de Galicia, 32900 Ourense, Spain; jmlorenzo@ceteca.net; 5 Preventive Medicine and Public Health, Food Sciencs, Toxicology and Forensic Medicine Department, University of Valencia, 91354 Valencia, Spain; francisco.barba@uv.es

**Keywords:** mushroom, antioxidant, shelf life, antimicrobial, sensory characteristics, natural extracts

## Abstract

The antioxidant and antimicrobial properties of *Cantharellus cibarius* decoction and the effect of mushroom addition on the physicochemical and microbiological properties of frankfurters during refrigerated storage were studied. Mushroom addition significantly reduced (*p* < 0.05) the formation of total aerobic mesophilic bacteria during storage. Regarding the texture, there was no negative effect in frankfurters with the mushroom added, compared to the control group of sausages. Generally, *C. cibarius* can be used as a natural ingredient in order to prevent the growth of microorganisms in cooked pork sausages, causing an extension in shelf life during chilled storage.

## 1. Introduction

Emulsification technology for frankfurter-type sausage production has been used over several hundred years. Frankfurters, as a type of emulsified meat product, do not have a permanent recipe, so their ratio of ingredients varies during production, and therefore these sausages are subjected to various undesirable changes [1]. Lipid oxidation is possibly the biggest problem in the storage of meat products and can cause negative changes that directly influence the product’s quality and its shelf life, as well as causing a change in color, taste, and deterioration [2]. In the food industry, traditional (synthetic) antioxidants are used to slow down the degradation process of the final product. However, more and more data are pointing out that these compounds can cause toxicity problems and negatively affect consumers’ health. A new way of preventing these effects is to use herbal antioxidants as the substitute for conventionally used antioxidants [3].

Edible mushrooms, whether dry or fresh, contain valuable compounds from a technological and nutritional aspect (proteins, phenolic compounds, and taste enhancers) [4]. Thus, they could be a potential source of natural antioxidants if incorporated into food products. In the previous studies, *Lentinus edodes* was added to frankfurters [5] and to fermented sausages [6], as well as *Agaricus bisporus* in cooked ground beef, but no reference has been found for the use of *Cantharellus cibarius* in cooked sausages.

Thus, the goal of this paper was to inspect the antioxidative and antimicrobial properties of *C. cibarius* decoction in vitro and in frankfurters, in order to examine the influence on the technological parameters, microbiological profile, and sensory quality during storage.

## 2. Material and Methods

### 2.1. Materials

Potassium persulfate, 2,2’-azino-bis (3-ethylbenzothiazoline-6-sulfonic acid) (ABTS), ferric chloride hexahydrate, tryptic Soy Agar (TSA), Tryptic Soy Broth (TSB), Yeast Malt Agar (YMA), Yeast Malt Broth (YMB), and 2,3,5-triphenyl tetrazolium chloride (TTC) were purchased from Sigma-Aldrich Chemical Co. (St. Louis, MO, USA), 96 well plates were procured from SARSTEDT AG & Co. KG (Nümbrecht, Germany), while ethanol was obtained from Reahem (Novi Sad, Serbia).

### 2.2. Sample Preparation

The powdered mushroom was bought as a commercial product from B.M.B. (Arilje, Serbia). In order to obtain a decoction, a mixture of dry powdered mushroom and Milli-Q water (Merck, Darmstadt, Germany) (1:10), was heated at 80 °C for 1 h. The resulting decoction was subsequently used for the evaluation of the antioxidant and antimicrobial capacity.

### 2.3. Antioxidant Activity of C. cibarius Decoction

The antioxidant activity was tested according to the Petrović et al. [7] method adapted for a 96-well microplate reader. The percentage of neutralization of the ABTS radical was calculated according to the formula:

(1) $$nABTS\;\left(\%\right)\;=\;\frac{\mathrm{Ablank}-\mathrm{Asample}}{\mathrm{Ablank}}\;\times \;100$$

L-ascorbic acid was used as the standard. All measurements were performed in triplicates.

### 2.4. Antimicrobial Activity Assay of C. cibarius Decoction

Three Gram positive bacterial strains—*Staphylococcus aureus* 25923, *Bacillus cereus* 11778, and *Listeria monocytogenes* 19111, three Gram negative bacterial strains—*Salmonella* Typhimurium 14028, *Escherichia coli* (O157:H7) 35150, and *Yersinia enterocolitica* 27729, as well as two yeasts—*Candida albicans* 10231 and *Pichia fermentans* 28789, were used to check the antimicrobial potential of *C. cibarius* decoction. All the selected bacteria and yeasts belonged to ATCC (American Type Culture Collection, Rockville, MD, USA).

The broth micro-dilution method was used to designate the minimum inhibitory (MIC) and minimum bactericidal/fungicidal (MBC/MFC) concentrations, as already explained earlier [8]. *C. cibarius* decoction concentrations in the range of 0.0097 to 20.0 mg/mL were used for the experiment.

### 2.5. Manufacturing of Frankfurters

The decoctions of *C. cibarius* were prepared as follows—60 g of powder was added to 2 L of distilled water and preheated at 80 °C. The decoctions obtained in this way were poured into a plastic container, cooled, and frozen in order to obtain ice. This was repeated in triplicate (T1). The same procedure was applied for the following three batches (T2), only with the exception that this time 120 g of powder was used in an individual batch. The control formulation (C) was prepared with ice obtained from distilled water (without mushrooms). All the treatments were formulated to obtain 8 kg batter (Table 1).

Fresh pork hams and pork back fat were bought from a local abattoir. All connective tissue and visible fat were removed from the ham muscles. Spice mixtures, primarily garlic and onions, were not used. Also, during the thermal treatment, smoke was not used for the same reason—to obtain the real antioxidant and antimicrobial effect of the decoction added to the sausages. After production, the frankfurters were kept in the refrigerated chamber (1–4 °C) for 60 days.

### 2.6. Proximate Composition Analyses

The protein (nitrogen × 6.25; ISO 937) [9], moisture (ISO 1442) [10], and total fat (ISO 1443) [11] contents of the frankfurters were calculated according to the recommended methods from the International Organization for Standardization. All the analyses were done in triplicate and using two technical replicates.

### 2.7. Color

The method was performed according to Tomasevic et al. [12] and the equipment used was calibrated according to the procedure explained in Tomasevic et al. [13]. The total color difference (Δ*E*) of each sample (S) was calculated with respect to the control sausage (C) using the following equation:

(2) $$\Delta E\;=\;\sqrt{\left(a_{S}^{*}-a_{C}^{*}\right)+{\left(b_{S}-b_{C}^{*}\right)}^{2}\;+\;{\left(L_{S}^{*}-L_{C}^{*}\right)}^{2}}$$

### 2.8. pH Measurement

The pH value was determined using the portable pH meter (Consort C931, Turnhout, Belgium) equipped with a piercing glass reinforced electrode (Mettler Toledo, Greifensee, Switzerland) for direct measuring of pH in meat products. The instrument was calibrated with standard phosphate buffers (pH was 7.02 and 4.00 at 20 °C). All the analyses were done in triplicate and using two technical replicates. 

### 2.9. Texture Profile Analyses (TPA)

The textural traits were carried out using the TA.XT Plus Texture Analyzer (Stable Micro Systems Ltd., Surrey, UK) with 5 kg load cell, at room temperature. The textural profile analyses (TPA) were performed using frankfurter cores from the middle (20 mm high and 12 mm diameter), positioned upright on a platform and compressed twice to 25% of their original height with a cylindrical aluminum probe (P/25). The pre-test speed and post-test speed were 180 mm min^−1^, while the test speed was 60 mm min^−1^. The attributes determined were—hardness, springiness, cohesiveness, and chewiness, according to Pons and Fiszman [14].

### 2.10. Microbological Analyses

In order to assess the microbiological safety of the frankfurter during storage in the refrigerator, determination of the total mesophillic, aerobic organisms—TPC, *Salmonella* spp., *Escherichia coli,* and *Listeria monocytogenes* was applied, according to the procedures described by the standards ISO 4833-2 [15], ISO 6579 [16], ISO 7251 [17], and ISO 11290-1 [18], respectively. The results were expressed as a log CFU/g. The microbiological analyses were performed in triplicate.

### 2.11. Sensory Evaluation

In total, 50 consumers from Belgrade, capital of Serbia, scored their liking for odor, taste, and overall quality of each type of sausage. A 9-point hedonic scale was used (1 = dislike extremely; 5 = neither like nor dislike; and 9 = like extremely) according to Peryam and Pilgrim [19].

### 2.12. Statistical Analyses

An analysis of variance (ANOVA) at *p* < 0.05 was done for statistical significance. The results of proximate composition, antioxidant properties in vitro, and MIC/MBC were subjected to the one-way ANOVA, Tukey’s HSD test. The other analyses were subjected to two-way ANOVA. The *C. cibarius* decoct concentration and storage time were set as independent variables and the readings of the results were set as the dependent variable. A Bonferroni test was conducted for the mean comparison at different levels of *C. cibarius* concentrations and storage days. All the data was analyzed using SPSS 17.0 (Chicago, IL, USA).

## 3. Results and Discussion 

### 3.1. ABTS Neutralization Activity of C. cibarius Decoction

**Figure 1:**
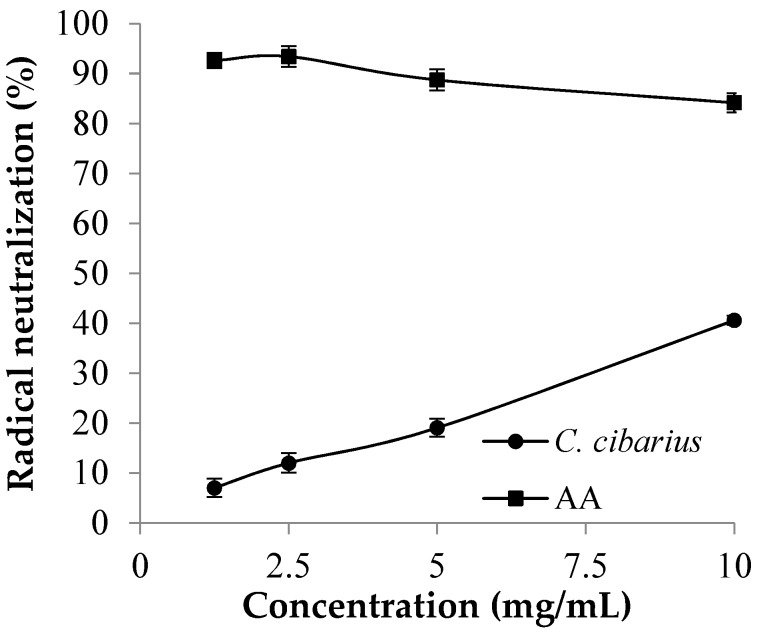
Antioxidative activity of *C. cibarius* decoction screened by the neutralization of ABTS radicals. Legend: Values are the arithmetic mean ± standard deviation (*n* = 3). ● = *Cantharellus cibarius* decoction (*C. cibarius*); ■ = commercial antioxidant, L-ascorbic acid (AA).

It was found that the decoct of *C. cibarius* exhibits neutralization of the ABTS radical in a dose-dependent manner (Figure 1). However, 50% of the radical neutralization had not been reached in the range of tested concentrations. Moreover, mushroom decoction was a weaker radical scavenger in comparison with ascorbic acid. Other authors found *C. cibarius* as a very potent source of antioxidative compounds, like polyphenols, ascorbic acid, and β-carotene [20,21]. The ability of the *C. cibarius* ethanol extract to scavenge ABTS radicals was tested by Dimitrijevic et al. [20], who reported an EC_50_ of about 14 mg/mL. In our study, the EC_50_ was a bit lower, around 12 mg/mL.

Having in mind that our sample was processed (dried) prior to the decoct preparation, and that other authors usually tested fresh samples, the results obtained in our study show a high antioxidant potency. Another restricting factor might be the material extraction method, which is simple cooking in water (decoction), instead of extraction. Water decoction or cooking in water is the simplest and the cheapest method for the extraction. In this study, the authors would like to test exactly this method due to its convenience (easy, fast, simple, and cheap) that would later easily find its way to industrial application. Thus, less amounts of non-polar compounds, like ergosterole and β-carotene, can be extracted and react with radicals.

### 3.2. Broth Microdilution Method

The antimicrobial property of *C. cibarius* decoction was studied against selected bacteria and yeasts, which are well known as foodborne pathogens (Table 2). The decoction has displayed significant antibacterial activity against *Yersinia enterocolitica* (MIC-10 mg/mL) and slight activity against *Listeria monocytogenes* at the highest concentration (MIC-20 mg/mL) tested. Regarding bactericidal activity, the decoction exhibited a desirable effect only at the highest concentration (20 mg/mL) towards *Listeria monocytogenes* and *Yersinia enterocolitica*. The fungistatic and fungicidal activity of *C. cibarius* decoction, against *Candida albicans*, was significantly better, expressed through the same value of MIC/MFC (10 mg/mL). Kosanic et al. [22] reported that acetonic and methanolic extracts of *C. cibarius* were active against *S. aureus* (MIC-5 and 10 mg/mL, respectively), *E. coli* (MIC-10 mg/mL for both extracts), and *C. albicans* (MIC-5 and 10 mg/mL, respectively). Also, Kozarski et al. [23] found that the antimicrobial effect of *C. cibarius* methanolic extracts, obtained with the same broth microdilution method, were active against *B*. *cereus* (MIC-0.3125 mg/mL), *L. monocytogenes* (MIC-0.625 mg/mL), *S. aureus* (MIC-1.25 mg/mL), *E. coli* O157:H7 (MIC-10 mg/mL), and *Y. enterocolitica* (MIC-20 mg/mL). Mushrooms are known as a valuable source of natural antibiotics [24]. More intense antimicrobial effects of mushrooms may occur due to the specific antimicrobial mechanisms of phenol, i.e., hydroxylation of the phenolic ring [25]. As several authors have reported, antimicrobial activity of the same species may vary depending on the origin of the mushroom, the solvent, and the bacterial strain [26].

Different species of the genus, *Candida*, accounting for less than 3% of the microbial population, originate from pastures, spreading via animal fur and gut on carcasses. In general, due to the newly created environmental conditions, ascomycetous yeasts become dominant over basidiomycetic yeasts during the processing of fresh meat and storage. The procedures used for the processing of fresh meat such as salting, drying, and fermentation (acidity and nitrates) favor the growth and development of *Candida* spp., including *C. albicans* [27].

Our study showed that *C. cibarius* decoction was most effective in inhibiting the growth/killing of *C. albicans*. Considering that this pathogenic yeast may be present not only during the various stages of processing fresh meat and refrigerated storage, but also in finished meat products, it seems that the addition of *C. cibarius* decoction would be highly desirable in protecting such products, e.g., frankfurter.

### 3.3. Proximate Composition, Color and pH

The results obtained with the addition of *C. cibarius* were at the normal ranges (Table 3) for these types of products [28,29].

The quality of meat is most commonly judged by consumers through the meat color [29]. Parameters of the color lightness (CIE L* value), redness (CIE a* value), and yellowness (CIE b* value) of examined sausages are shown in Table 4. In the present study, the incorporation of *C. cibarius* caused a kind of lower, but statistically significant (*p* < 0.05) the L* (lightness) value of frankfurter compared with the control, on day 1 of examination. T1 and T2 had higher values for yellowness than C. The decline in the lightness and the rise in yellowness presumably results from the suppression of the product’s natural color (fallow) owing to the addition of mushrooms. Similar results for lightness and yellowness was reported by Pil-Nam et al. [5], who added shiitake powder in frankfurters. The T2 treatment were generally “redder” than T1 and C. Taking into consideration the total color difference (Δ*E*) between frankfurters with added mushroom with respect to the control samples (at day 1), both values were lower than 3. Δ*E* is the difference between two colors in L* a* b* color space. Fernández-López et al. [30] stated that only Δ*E* higher than 3 CIELAB units would be differentiated by an observer. When likened to the addition of 0.5–5% grape seed flour, or sunflower seed oil/makgeolli lees fiber mixture, the result was a decline in lightness, redness, and yellowness of a similar product [31,32]. Therefore, the addition of *C. cibarius* has a trivial influence on the color characteristics of this product.

During the storage time, all the treatments increased in redness. In parallel, all the frankfurters decreased in lightness after 60 days of storage. Regarding the yellowness, there was decrease during storage, but on the 60th day of storage, it showed a statistically significant increase compared to the 1st day after the production of the frankfurters. Similar results for the yellowness during the storage time were provided by Fernández-López et al. [30]. Throughout the storage, all the color differences (Δ*E*) were increased, all of them being higher than 3.

The pH values are presented in Table 4. The obtained results were inside the usual ranges for this kind of meat product [33]. On the 60th day of storage, there was a decline in pH values compared to the first day of storage, but it was only statistically significant in T1. The decrease in pH value throughout the storage is presumably an aftereffect of the creation of organic acids from carbohydrates, produced by lactic acid bacteria [34]. Related results and the decline of pH values in frankfurters at the end of the storage period were reported by Ranucci et al. [35].

### 3.4. Texture Analyses

The textural traits of sausages are displayed in Table 5. On the first day after production, there was no difference (*p* > 0.05) in hardness, springiness, cohesiveness, and chewiness between the control group and *C. cibarius* frankfurters. Additionally, there was no statistically significant difference (*p* > 0.05) in the springiness and cohesiveness between T1, T2, and C at all days of examination. Regarding the hardness and chewiness, the sausages with added mushroom had higher values than the control group, at all days of examination during the storage. The storage time did not significantly (*p* > 0.05) influence the hardness, springiness, and chewiness within all the variants of frankfurters between the 1st to 60th days of storage, except the control group on the 60th day for chewiness. Almost identical trends were reported by Pil-Nam et al. [5], namely, hardness and chewiness were similar between the first and last days of storage, after the addition of *L. edodes* in frankfurters. On the other hand, the cohesiveness all of the three treatments showed a lower, but statistically significant (*p* < 0.05) increase during the storage. These findings indicate that although *C. cibarius* was added to sausages in different ratios, the textural traits changed only slightly.

### 3.5. Microbiological Analyses during Storage

The storage stability of the food, as well as meat products in general, is usually reflected by the number of aerobic mesophilic bacteria [6]. The TPC in frankfurters produced with *C. cibarius* decoction was significantly lower (*p* < 0.05) than that found in the control on the first day of storage (Table 6). It was probably the consequence of mushroom decoction addition. Namely, it is well known that the antimicrobial effect of mushrooms can be accomplished due to the presence of certain antimicrobial compounds, above all catechin, gallic acid, and rutin [36]. All treatments of the studied sausages increased the number of TPC. Similar results were reported by Tahmouzi et al. [37]. A higher increase in TPC was noticed for the control, whereas lower for the treatments with mushroom decoction added, except on the 40th day. On the 60th day of storage, the TPC (T1, T2) in the present study were lower, compared to the levels in the same product type enriched with *L. edodes* on the 30th day of storage as reported by Pil-Nam et al. [5]. In all three groups of sausages, *Salmonella* spp., *E. coli,* and *L. monocytogenes* were not detected.

### 3.6. Sensory Analyses

The sensory attributes of the frankfurters during chilled storage are presented in Table 7. The highest odor scores were obtained for T2, except for the 40th day of examination, where C obtained better estimates. However, no statistically significant difference (*p* > 0.05) between C and T1, T2 were found in the odor. Concerning the taste, the highest scores were given to the treatments with mushroom added on the 1st, 10th, 20th, and 30th day of examination. On the 40th, 50th, and 60th day of the examination, higher marks were given to the control group of frankfurters. There were no differences between C and T1, T2 in the taste (*p* > 0.05). The panelists marked the taste and odor of *C. cibarius* frankfurters with higher scores due to the fact that this mushroom contains particular compounds characterized as flavor enhancers (lenthionine and monosodium glutamate). The overall quality analyses showed a similar trend to that of taste. There was also no statistically significant difference between C and T1, T2 for the overall quality (*p* > 0.05). Regarding the storage effect, there was no statistically significant difference (*p* > 0.05) within the control group and *C. cibarius* sausages from the 1st to 60th day of storage, except for T2 for the overall quality.

Özvural and Vural [32] showed that the addition of grape flour powder resulted in lower estimates of taste. As well, Deda et al. [38] displayed that the tomato paste added in frankfurters caused off-taste alterations. In comparison to previously mentioned studies, the utilization of *C. cibarius* did not cause negative effects on the sensory traits, inversely, it improved the odor, taste, and overall acceptability during the first month of cold storage.

## 4. Conclusions

Sausages with mushroom added increased the shelf life by reducing the number of TPC during chilled storage. Frankfurters produced with *C. cibarius* had a partial improvement in the estimates for odor, taste, and overall quality, compared to the control group. Considering all the results of this research, hot water extract, or *C. cibarius* decoction, possesses certain antioxidative and antimicrobial potential and could be used in the production of frankfurters, to potentially prevent lipid oxidation and microorganisms growth in the final product.

## Figures and Tables

**Table 1:** Formula of the different types of sausages (expressed as % of the different ingredients in the formula).

Ingredients	T1	T2	C
Meat	48%	48%	48%
Fat	25%	25%	25%
Ice	-	-	25%
Decoction 1	25%	-	-
Decoction 2	-	25%	-
Sodium nitrite	1.7%	1.7%	1.7%
Polyphosphate	0.3%	0.3%	0.3%

T1 = concentration of 0.75% *C. cibarius* in the batch; T2 = concentration of 1.5% *C. cibarius* in the batch; C = control, ice instead *C. cibarius* decoction. “-” means not added in the preparation formula of frankfurters

**Table 2:** Antimicrobial activity of *C. cibarius* decoction, determined by the broth microdilution method, expressed as MIC (mg/mL) and MBC/MFC (mg /mL).

Bacterial Strain	Source		Concentration (mg/mL)
*Staphylococcus aureus*	ATCC 25923	MIC	>20
		MBC	>20
*Bacillus cereus*	ATCC 11778	MIC	>20
		MBC	>20
*Listeria monocytogenes*	ATCC 19111	MIC	20 ^A^
		MBC	20 ^A^
*Salmonella* Typhimurium	ATCC 14028	MIC	>20
		MBC	>20
*Escherichia coli* (0157:H7)	ATCC 35150	MIC	>20
		MBC	>20
*Yersinia enterocolitica*	ATCC 27729	MIC	10 ^B^
		MBC	20 ^A^
*Candida albicans*	ATCC 10231	MIC	10 ^B^
		MFC	10 ^B^
*Pichia fermentans*	ATCC 28789	MIC	>20
		MFC	>20

As there was no difference between the measurements (*n* = 3), standard deviations were not shown. Within the same column, means followed by different uppercase letters were significantly different (*p <* 0.05).

**Table 3:** Proximate analysis of frankfurters (day 1).

Parameter	T1	T2	C
Fat	25.75 ± 0.13 ^a^	25.85 ± 0.27 ^a^	29.31 ± 1.21 ^b^
Moisture	56.63 ± 1.06 ^a^	55.03 ± 0.81 ^b^	57.66 ± 1.22 ^a^
Protein	12.32 ± 0.46 ^a^	12.38 ± 0.6 ^a^	10.91 ± 0.17 ^b^

T1 = concentration of 0.75% *C. cibarius* in the batch; T2 = concentration of 1.5% *C. cibarius* in the batch; C = control, ice instead *C. cibarius* decoction. Values are the arithmetic mean ± standard deviation (*n* = 3 samples from each group × 2 replications). Different letters in the same row indicate significant differences (*p* < 0.05).

**Table 4:** CIELAB color coordinates (L*, a* and b*), color differences (ΔE*) respect to control sample and evolution of pH during storage of frankfurters.

Parameter	Storage Time (Days)	T1	T2	C
CIE L*	1	75.03 ± 1.71 ^a,A^	72.11 ± 2.08 ^b,A^	75.63 ± 1.88 ^c,A^
	20	75.01 ± 1.46 ^a,A^	70.22 ± 1.93 ^b,B^	75.29 ± 5.39 ^a,A,B^
	40	73.01 ± 1.98 ^a,B^	69.34 ± 1.72 ^b,C^	75.25 ± 1.42 ^c,A^
	60	64.59 ±4.06 ^a,C^	59.7 ± 3.68 ^b,D^	74.73 ± 1.39 ^c,B^
CIE a*	1	7.49 ± 0.92 ^a,A^	8.9 ± 0.91 ^b,A^	7.45 ± 0.82 ^a,A^
	20	8.09 ± 0.73 ^a,B^	9.65 ± 0.85 ^b,B^	8.5 ± 0.76 ^c,B^
	40	9.1 ± 0.87 ^a,C^	10.43 ± 0.9 ^b,C^	9.43 ± 1.02 ^c,C^
	60	9.76 ± 0.76 ^a,D^	11.1 ± 1.19 ^a,D^	9.98 ± 0.82 ^a,D^
CIE b*	1	6.42 ± 0.93 ^a,A^	8.27 ± 1.01 ^b,A,C^	4.14 ± 0.71 ^c,A^
	20	5.48 ± 0.78 ^a,B^	8.38 ± 0.94 ^b,A^	3.84 ± 0.61 ^c,B^
	40	6.32 ± 0.89 ^a,A^	8.08 ± 1.04 ^b,C^	3.32 ± 0.55 ^c,C^
	60	7.17 ± 1.05 ^a,C^	9.63 ± 0.93 ^b,B^	4.63 ± 0.83 ^c,D^
Δ*E*	1	2.35 ± 0.96 ^a,A^	2.95 ± 0.09 ^b,A^	-
	20	1.8 ± 0.12 ^a,B^	6.90 ± 0.66 ^b,B^	-
	40	3.75 ± 0.11 ^a,C^	7.65 ± 0.47 ^b,C^	-
	60	10.45 ± 0.59 ^a,D^	15.47 ± 0.83 ^b,D^	-
pH	1	6.16 ± 0.01 ^a,A^	6.11 ± 0.01 ^b,A^	6.17 ± 0.02 ^a,A,B^
	20	6.15 ± 0.01 ^a,A,B^	6.11 ± 0.01 ^b,A^	6.19 ± 0.02 ^c,A^
	40	6.10 ± 0.04 ^a,A,B^	6.09 ± 0.02 ^a,A^	6.18 ± 0.05 ^b,A,B^
	60	6.07 ± 0.03 ^a,B^	6.05 ± 0.02 ^a,A^	6.11 ± 0.06 ^a,B^

T1 = concentration of 0.75% *C. cibarius* in the batch; T2 = concentration of 1.5% *C. cibarius* in the batch; C = control, ice instead *C*. *cibarius* decoction. Values are the arithmetic mean ± standard deviation. Color: *n* = 3 samples from each group × 5 points on the cutting surface. pH: *n* = 3 samples from each group × 2 replications. Values with different lowercase letters (a–c) in the same row differ significantly (*p* < 0.05). Values with different uppercase letters (A–D) in the same column differ significantly (*p* < 0.05).

**Table 5:** Texture profile analysis (TPA) parameters of frankfurters during storage.

Texture Profile Parameters	Storage Time (Days)	T1	T2	C
Hardness (N)	1	1.99 ± 0.28 ^a,A,B^	2.00 ± 0.21 ^a,A,C^	1.98 ± 0.32 ^a,A^
	10	2.29 ± 0.19 ^a,A^	2.55 ± 0.27 ^a,B^	1.82 ± 0.34 ^b,A^
	20	2.02 ± 0.14 ^a,A,B^	2.09 ± 0.16 ^a,A,C^	1.73 ± 0.12 ^b,A^
	30	1.90 ± 0.23 ^a,A,B^	2.10 ± 0.22 ^a,A,C^	1.68 ± 0.12 ^b,A^
	40	1.82 ± 0.12 ^a,B^	2.36 ± 0.16 ^b,A,B^	1.62 ± 0.11 ^c,A^
	50	1.88 ± 0.12 ^ab,A,B^	2.16 ± 0.35 ^a,A,B,C^	1.78 ± 0.26 ^b,A^
	60	1.95 ± 0.14 ^a,A,B^	1.99 ± 0.23 ^a,C^	1.60 ± 0.20 ^b,A^
Springiness	1	0.96 ± 0.03	0.95 ± 0.03	0.96 ± 0.02
	10	0.94 ± 0.04	0.93 ± 0.03	0.95 ± 0.03
	20	0.96 ± 0.02	0.94 ± 0.03	0.95 ± 0.03
	30	0.95 ± 0.01	0.96 ± 0.02	0.95 ± 0.02
	40	0.96 ± 0.02	0.94 ± 0.02	0.94 ± 0.02
	50	0.95 ± 0.03	0.96 ± 0.01	0.96 ± 0.02
	60	0.95 ± 0.02	0.93 ± 0.05	1.08 ± 0.31
Cohesiveness	1	0.84 ± 0.02 ^A^	0.83 ± 0.02 ^A^	0.83 ± 0.02 ^A^
	10	0.84 ± 0.01 ^A^	0.83 ± 0.01 ^A^	0.84 ± 0.01 ^A^
	20	0.84 ± 0.02 ^A^	0.84 ± 0.01 ^A,B^	0.83 ± 0.03 ^A^
	30	0.85 ± 0.01 ^A,B^	0.84 ± 0.01 ^A,B^	0.82 ± 0.05 ^A,B^
	40	0.85 ± 0.01 ^A,B^	0.84 ± 0.02 ^A,B^	0.84 ± 0.02 ^A,B^
	50	0.86 ± 0.02 ^B,C^	0.85 ± 0.01 ^B,C^	0.86 ± 0.03 ^B^
	60	0.87 ± 0.03 ^B,C^	0.87 ± 0.03 ^B,C^	0.86 ± 0.02 ^B^
Chewiness (N)	1	1.63 ± 0.20 ^a,A^	1.68 ± 0.24 ^a,A^	1.62 ± 0.25 ^a,A^
	10	1.83 ± 0.17 ^ab,A^	2.07 ± 0.26 ^a,B,C^	1.59 ± 0.23 ^b,A^
	20	1.72 ± 0.17 ^a,A^	1.78 ± 0.15 ^a,A,B^	1.61 ± 0.21 ^a,A^
	30	1.81 ± 0.19 ^a,A^	1.96 ± 0.19 ^a,A,B,C^	1.32 ± 0.15 ^b,A^
	40	1.65 ± 0.07 ^a,A^	2.13 ± 0.19 ^b,C^	1.25 ± 0.07 ^c,B^
	50	1.66 ± 0.16 ^a,A^	1.89 ± 0.16 ^b,A,B,C^	1.05 ± 0.15 ^c,B^
	60	1.66 ± 0.12 ^a,A^	1.74 ± 0.12 ^a,A,B^	1.09 ± 0.17 ^b,B^

T1 = concentration of 0.75% *C. cibarius* in the batch; T2 = concentration of 1.5% *C. cibarius* in the batch; C = control, ice instead C. cibarius decoction. Values are the arithmetic mean ± standard deviation (*n* = 8 specimens × 3 samples from each group). Values with different lowercase letters (a–c) in the same row differ significantly (*p <* 0.05). Values with different uppercase letters (A–C) in the same column differ significantly (*p <* 0.05).

**Table 6:** Microbiological profile of frankfurters during refrigerated storage.

Microorganisms	Storage Time (days)	T1	T2	C
(TPC) (log_10_ cfu/g)	1	1 ± 0 ^a,A^	1.26 ± 0.24 ^a,A^	2.23 ± 0.21 ^b,A^
	20	4.8 ± 0.17 ^a,B^	4.36 ± 0.1 ^b,B^	5.76 ± 0.06 ^c,B^
	40	4.88 ± 0.03 ^a,B^	5.5 ± 0.03 ^b,C^	4.41 ± 0.11 ^c,C^
	60	5.05 ± 0.02 ^a,B^	4.7 ± 0.1 ^b,B^	5.93 ± 0.08 ^c,B^
*Salmonella* spp.	1	ND	ND	ND
	20	ND	ND	ND
	40	ND	ND	ND
	60	ND	ND	ND
*E. coli*	1	ND	ND	ND
	20	ND	ND	ND
	40	ND	ND	ND
	60	ND	ND	ND
*L. monocytogenes*	1	ND	ND	ND
	20	ND	ND	ND
	40	ND	ND	ND
	60	ND	ND	ND

T1 = concentration of 0.75% *C. cibarius* in the batch; T2 = concentration of 1.5% *C. cibarius* in the batch; C = control, ice instead C. cibarius decoction. Values are the arithmetic mean ± standard deviation (*n* = 3 samples from each group × 2 replications). Values with different lowercase letters (a–c) in the same row differ significantly (*p <* 0.05). Values with different uppercase letters (A–D) in the same column differ significantly (*p <* 0.05). ND = not detected.

**Table 7:** Sensory characteristics of frankfurters during storage.

Sensory Quality Parameters	Storage Time (Days)	T1	T2	C
Odor	1	6.1 ± 2.1 ^A,B^	6.3 ± 2.2	5.6 ± 2.4 ^A^
	10	6.8 ± 1.8 ^A^	7.0 ± 1.7	6.5 ± 2.0 ^A,B^
	20	7.1 ± 1.8 ^A^	7.3 ± 1.8	6.7 ± 1.9 ^A,B^
	30	6.6 ± 2.1 ^A,B^	6.8 ± 2.1	6.8 ± 1.8 ^B^
	40	6.3 ± 2.1 ^A,B^	6.7 ± 2.0	7.0 ± 2.1 ^B^
	50	6.0 ± 1.9 ^A,B^	6.4 ± 1.8	6.3 ± 1.9 ^A,B^
	60	5.6 ± 1.1 ^B^	6.4 ± 1.2	6.1 ± 1.5 ^A,B^
Taste	1	6.5 ± 2.1 ^A,C,D^	7.1 ± 2.1 ^A,B^	6.4 ± 2.2 ^A^
	10	7.6 ± 1.6 ^B^	7.8 ± 1.5 ^A^	7.5 ± 1.6 ^B,C^
	20	7.7 ± 1.5 ^B^	7.8 ± 1.5 ^A^	7.5 ± 1.8 ^B,C^
	30	7.4 ± 1.8 ^A,B^	6.9 ± 2.3 ^A,B,C^	7.3 ± 1.4 ^B,C^
	40	7.0 ± 1.9 ^A,B,C^	7.1 ± 1.9 ^A,B^	7.6 ± 1.8 ^C^
	50	6.2 ± 1.9 ^C,D^	6.5 ± 1.7 ^B,C^	6.6 ± 2.0 ^A,B^
	60	5.9 ± 1.2 ^D^	5.9 ± 1.4 ^C^	6.5 ± 1.4 ^A^
Overall quality	1	6.5 ± 2.1 ^A,C^	6.9 ± 2.1 ^A^	6.2 ± 2.1 ^A^
	10	7.4 ± 1.5 ^A,B^	7.8 ± 1.5 ^A^	7.4 ± 1.5 ^B^
	20	7.6 ± 1.4 ^B^	7.7 ± 1.5 ^A^	7.4 ± 1.7 ^B^
	30	7.4 ± 1.8 ^A,B^	7.4 ± 2.2 ^A^	7.3 ± 1.4 ^B^
	40	6.9 ± 2.0 ^A,B,C^	7.0 ± 2.0 ^A^	7.4 ± 2.0 ^B^
	50	6.5 ± 2.0 ^A,C^	6.8 ± 1.6 ^A,B^	6.9 ± 2.0 ^A,B^
	60	6.0 ± 1.1 ^C^	5.9 ± 1.3 ^B^	6.5 ± 1.5 ^A,B^

T1 = concentration of 0.75% *C. cibarius* in the batch; T2 = concentration of 1.5% *C. cibarius* in the batch; C = control, ice instead C. cibarius decoction. Values are the arithmetic mean ± standard deviation (*n* = 50 assessors on each day of examination). Values with different lowercase letters (a–c) in the same row differ significantly (*p <* 0.05). Values with different uppercase letters (A–D) in the same column differ significantly (*p <* 0.05).
